# Genotyping-in-Thousands by sequencing panel development and application for high-resolution monitoring of introgressive hybridization within sockeye salmon

**DOI:** 10.1038/s41598-022-07309-x

**Published:** 2022-03-02

**Authors:** Sarah L. Chang, Hillary G. M. Ward, Lucas D. Elliott, Michael A. Russello

**Affiliations:** 1 Department of Biology, University of British Columbia, Kelowna, BC Canada; 2 Lands and Natural Resource Operations and Rural Development, British Columbia Ministry of Forests, Penticton, BC Canada; 3 UiT The Arctic University of Norway, Tromsø, Norway

**Keywords:** Molecular ecology, Genetic hybridization

## Abstract

Stocking programs have been widely implemented to re-establish extirpated fish species to their historical ranges; when employed in species with complex life histories, such management activities should include careful consideration of resulting hybridization dynamics with resident stocks and corresponding outcomes on recovery initiatives. Genetic monitoring can be instrumental for quantifying the extent of introgression over time, however conventional markers typically have limited power for the identification of advanced hybrid classes, especially at the intra-specific level. Here, we demonstrate a workflow for developing, evaluating and deploying a Genotyping-in-Thousands by Sequencing (GT-seq) SNP panel with the power to detect advanced hybrid classes to assess the extent and trajectory of intra-specific hybridization, using the sockeye salmon (*Oncorhynchus nerka*) stocking program in Skaha Lake, British Columbia as a case study. Previous analyses detected significant levels of hybridization between the anadromous (sockeye) and freshwater resident (kokanee) forms of *O. nerka*, but were restricted to assigning individuals to pure-stock or “hybrid”. Simulation analyses indicated our GT-seq panel had high accuracy, efficiency and power (> 94.5%) of assignment to pure-stock sockeye salmon/kokanee, F_1_, F_2_, and B_2_ backcross-sockeye/kokanee. Re-analysis of 2016/2017 spawners previously analyzed using TaqMan® assays and otolith microchemistry revealed shifts in assignment of some hybrids to adjacent pure-stock or B_2_ backcross classes, while new assignment of 2019 spawners revealed hybrids comprised 31% of the population, ~ 74% of which were B_2_ backcross or F_2_. Overall, the GT-seq panel development workflow presented here could be applied to virtually any system where genetic stock identification and intra-specific hybridization are important management parameters.

## Introduction

Global fish populations are in decline due to agricultural development, rapidly rising riverine water temperatures, and reduced connectivity^1–6^. In particular, 80% of salmonid populations in the Columbia River have experienced losses linked to hydroelectric development and the destruction of spawning and rearing habitat^7,8^. In response, management strategies have included incorporating fish passage at dams to mitigate impacts to migratory fish life histories and supplementing populations in decline with hatchery fry for stock enhancement. For example, Coho salmon (*Oncorhynchus kisutch*) have been restored in the lower Columbia River using hatchery stocking, resulting in the establishment of local naturalized populations^5,9^. Additionally, the enhancement of spawning habitat has been an effective strategy to restore fish populations, where the replacement of river substrate improved water velocities, dissolved oxygen, and usage of the site by spawning Chinook salmon (*O. tshawytscha*)^10^. Ultimately, the success of fish stocking programs that target species with diverse migratory and resident forms should include careful consideration of resulting hybridization dynamics and corresponding outcomes on recovery initiatives.

Hybridization in fish species is well documented, including inter-specific hybrids such as rainbow (*Oncorhynchus mykiss*) and cutthroat trout (*O. clarkii*)^11^, as well as within-species hybrids between different life history forms, such as the case between anadromous sockeye salmon and freshwater resident kokanee^12^. The long-term effects of hybridization are often complex, with the potential for both positive and negative outcomes^13^. Hybridization can be a powerful conservation tool harnessed to rescue populations with low genetic diversity and increase fitness through the integration of favorable traits such as larger body size, more offspring, and longer lifespans^13–16^. On the other hand, detrimental effects have been observed such as introgression with maladapted gene complexes^17^, decrease in reproductive success^18^, and negative impacts on growth^19^, with hybrid fitness theorized to decrease as the divergence between parental phenotypes increases^20^. Overall, the impacts of intra-specific hybridization in the wild are still not well understood, warranting further study, especially when observed as part of an active management program.

*Oncorhynchus nerka* provides an excellent system for investigating the genetic and physiological outcomes of hybridization as it exhibits tremendous life history variation, shows natal homing behavior, and represents a valuable species targeted for population restoration through restocking^21^. This species exhibits two main migratory forms, including anadromous sockeye salmon (hereafter referred to as “sockeye salmon”) and freshwater resident kokanee (hereafter referred to as “kokanee”). Kokanee are much smaller than sockeye salmon (26 cm versus > 45 cm average adult fork length) and occur sympatrically in many lakes, but tend to exhibit different spawning habitat preferences and spawning periods^22,23^. Despite differences in spawning behavior, kokanee males are known to sneak on spawning sockeye salmon females^24^, and size-selective mating has been observed between male sockeye salmon and female kokanee^25^, allowing for gene flow between migratory forms. Sockeye-kokanee hybridization can lead to an increase in body size of resident hybrids that can increase angler satisfaction^19^ and bolster the overall genetic diversity of *O. nerka* in the system^16^. Conversely, hybrids can also experience negative impacts such as lower swimming capabilities than pure sockeye salmon^26^, medial seawater adaptabilities^27^, intermediate maturation time^23^, decreased egg survival rate^28^, and the loss of the iconic red coloration that is key in sexual selection^25^. With a broad range of potential outcomes, accurate assessment of the extent of introgression is critical for elucidating the long-term effects of hybridization associated with supplementation and reintroduction programs.

To address large-scale declines in sockeye salmon populations in the Columbia River, an experimental reintroduction program was initiated in Skaha Lake within the Okanagan Basin of the Southern Interior of British Columbia, Canada. This program provides an excellent opportunity to investigate the extent and outcomes of intra-specific hybridization between *O. nerka* migratory and resident forms outside of a laboratory setting. Previous research examining hybridization between sockeye salmon and kokanee found *O. nerka* hybrids occupy intermediate morphologies and exhibit a largely resident life history, although this latter finding requires further investigation^12,29^. These studies employed genetic marker sets that were effective at differentiating pure-stock from hybrid, however, they had limited power for the identification of advanced hybrid classes that has become increasingly important for genetic monitoring as the reintroduction program matures. The detection of advanced hybrid classes within a species can be challenging due to the proximity of backcrosses to pure genotype frequencies. For example, allozyme loci were only able to reveal intermediate allelic frequencies suggesting hybridization between transplanted and native sockeye^30^, while microsatellite assays have the ability to detect introgression to the F_1_ level^31^, but both marker types experience difficulty in the detection of advanced hybrid classes based on the conventional number of loci employed^32,33^. In that regard, traditional markers may not have the statistical power to identify advanced hybrid classes^34^, with an estimated minimum of 70 markers required to discriminate between pure parental species and advanced backcrosses^35^. A previous simulation-based sensitivity analysis found that a panel comprised of 300 highly differentiated SNPs has the resolution needed to detect advanced hybrid classes between sockeye salmon and kokanee in Skaha Lake^36^. With this in mind, advancements in massively parallel sequencing can be leveraged to improve the identification of hybridization in systems by pooling barcoded amplicons to increase the power of genetic panels for classification^37^; Genotyping-in-Thousands by sequencing (GT-seq) is a particularly useful approach in cases where large sample sizes need to be cost-effectively genotyped^38^.

Here, we demonstrate a workflow for developing, evaluating and deploying a GT-seq SNP panel with the power to detect advanced hybrid classes to assess the extent and trajectory of intra-specific hybridization, using the sockeye salmon reintroduction program in the Okanagan Basin as a case study. Using previously published restriction site associated DNA sequencing (RAD-seq) collected for this system, we first simulated two pure (kokanee, sockeye) and four hybrid classes (F_1_, F_2_, B_2_ backcross-kokanee, B_2_ backcross-sockeye) with three different SNP datasets (300 highest *F*_*st*_ SNPs; 600 highest *F*_*st*_ SNPs; 350 random SNPs selected from the 600 highest *F*_*st*_ SNPs) to assess information content to inform panel construction. We then evaluated the accuracy, efficiency, and power of the optimized GT-seq panel using simulation analyses. We further assessed panel performance by re-genotyping individuals sampled in Skaha Lake in 2016 and 2017 that were previously genetically assigned as pure-stock or F_1_ hybrid at 32 SNPs and subjected to otolith microchemistry analysis to reconstruct migratory history^29^. Finally, we genetically assigned *O. nerka* spawners sampled in 2019 to pure-stock or hybrid class (F_1_, F_2_, B_2_ backcross-kokanee, B_2_ backcross-sockeye) to estimate stock proportions and examine trends in this system over time.

## Methods

### Study system and samples

**Figure 1:**
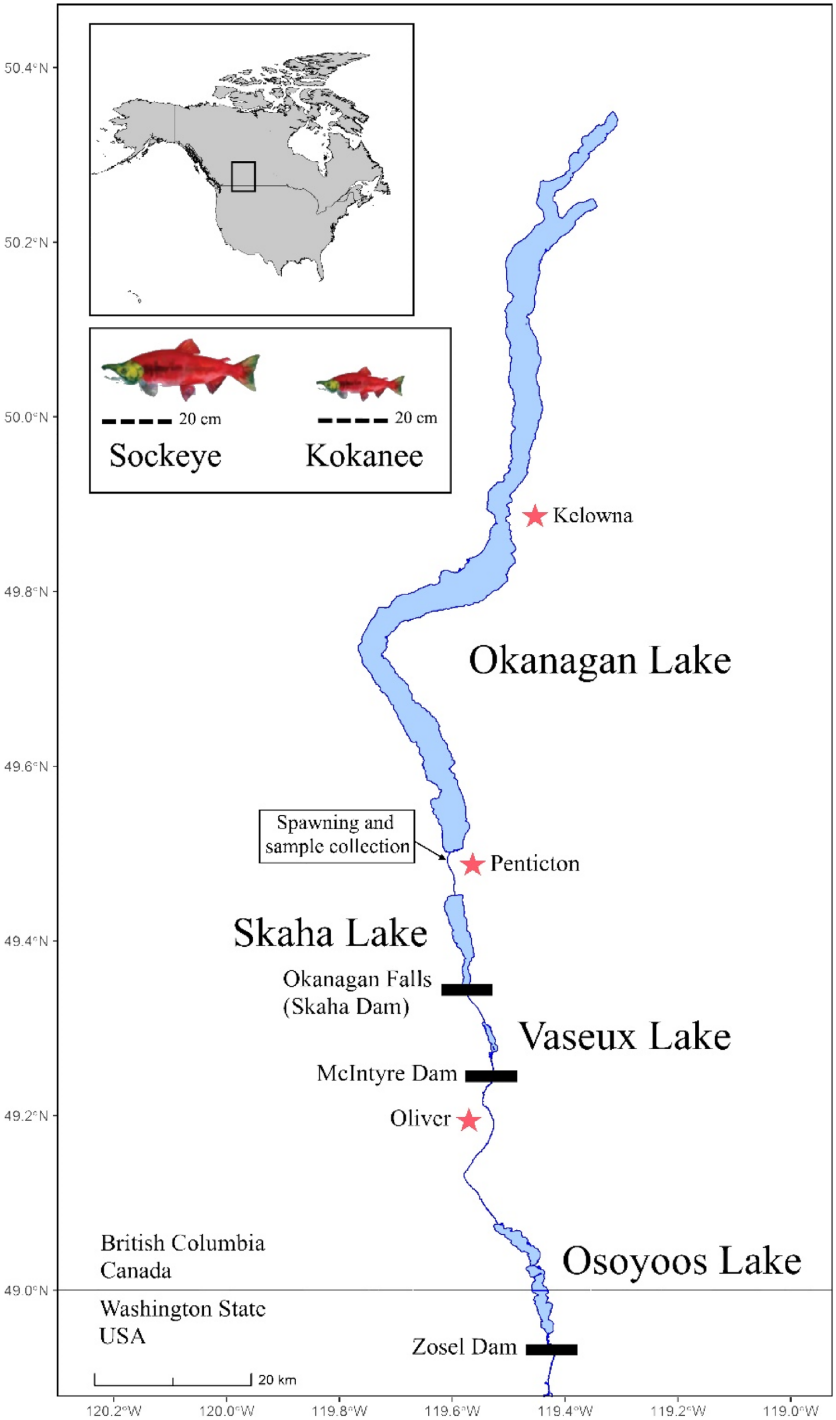
Map of the Columbia River displaying Skaha Lake, Okanagan River (extending southward originating at McIntyre Dam), and downstream lakes and dams. Size comparison of an average sockeye male and kokanee male included. The maps were created using a custom R script (https://github.com/changsarahl/BCmap) with R version 4.1.1 (https://www.R-project.org/). Geographic data were accessed under the open government license—British Columbia: https://catalogue.data.gov.bc.ca/dataset/freshwater-atlas-watersheds and the Washington geospatial open data license: https://geo.wa.gov/datasets/waecy::wa-hydrography-nhdwaterbody/about.

Skaha Lake is located in the Canadian portion of the Okanagan Basin, and flows south to join the Columbia River (Fig. 1). *O. nerka* spawn in the Okanagan River, upstream of Skaha Lake. Historically, sockeye salmon populations existed in the Okanagan Basin, however, the construction of a dam at McIntyre Bluff in 1921 blocked access to spawning grounds and channelization of the Okanagan River further degraded spawning habitat. These activities left Skaha Lake with a sole population of stream-spawning kokanee^39^. In attempts to restore sockeye salmon to the Okanagan Basin, a re-introduction program was implemented in 2004. Through this initiative, upstream fish passage was created by making structural improvements to migration barriers, restoring stream habitat, and stocking sockeye salmon fry annually into Skaha Lake. This system now supports a self-sustaining population of sockeye salmon^39,40^.

We genotyped tissue samples from *O. nerka* spawners in the Okanagan River upstream of Skaha Lake that were previously sampled in 2016 (n = 45) and 2017 (n = 59)^29^, and newly sampled in 2019 (n = 218; this study) as deadpitch (post-mortem carcass collection after spawning) by personnel from the BC Ministry of Forests, Lands, Natural Resource Operations, and Rural Development. The section of river that was sampled represents the only spawning habitat that exists for *O. nerka* populations from Skaha Lake and all forms spawn within this section of river. Samples were collected on multiple dates that spanned the duration of the spawning run. Biological data (length and sex) and tissue samples (operculum punches preserved in ethanol) were collected from all sampled fish. Moreover, the 2016 and 2017 samples were previously genetically assigned to pure-stock or F_1_ hybrid using a panel of 32 TaqMan® SNP assays and subjected to otolith microchemistry analysis^36^ to determine fish migratory history and investigate maternal migratory signatures that are passed transgenerationally to offspring^41^.

### GT-seq SNP panel design

We used previously published genotypic data collected via RAD-seq^42^ for: (1) Skaha Lake kokanee (n = 20) collected in 2003 prior to the sockeye salmon restocking program; and (2) Okanagan River sockeye salmon (n = 35) collected in 2012 downstream of historical migration barriers. Using the *populations* module in STACKS version 2.0 beta 8^43^, we required all loci to be present in at least 60% of individuals in both populations, with a minor allele frequency greater than 0.05. Due to the salmonid whole genome duplication event, we filtered out suspected homeologs by removing any locus with a negative F_*is*_ or H_*obs*_ > 0.5 that occurred in both reference populations^44^. The resulting SNP dataset was then filtered for quality using VCFtools^45^ to remove SNPs not in Hardy–Weinberg equilibrium and to calculate Weir and Cockerham (1984)’s *θ*, an unbiased estimate of *F*_*st*_^46^, between Skaha Lake kokanee and Okanagan River sockeye salmon following^36^. We removed loci with insufficient flanking sequence required for primer design by retaining loci with the SNP positioned between the 40th and 70th base pairs of the RAD tag sequences and selected the top 650 loci exhibiting the highest *F*_*st*_. We then assessed all loci pairs for deviation from linkage equilibrium using GENEPOP 4.5^47^, removed loci that were linked, and finalized selection of 600 candidate loci.

To examine panel informativeness of candidate loci and forecasting of panel ability after primer dropout, we simulated parental and offspring individuals (n = 1000) for six hybrid classes (kokanee, sockeye, F_1_, F_2_, B_2_ backcross-kokanee, B_2_ backcross-sockeye) with *recom-sim.py* (https://github.com/salanova-elliott/recom-sim). Separate simulations were conducted at: 1) 300 highest *F*_*st*_ SNPs; 2) 600 highest *F*_*st*_ SNPs; and 3) 350 SNPs randomly selected from the 600 highest *F*_*st*_ SNPs. We assigned simulated individuals to parental or hybrid class by calculating the posterior probabilities of membership as implemented in NEWHYBRIDS^48^ with the reference populations flagged as known genotypes with the “*z*” option, and constructed confusion matrices for each panel assessment. After *in-silico* assessment, we sent the full RAD tag sequences that were associated with the pool of candidate SNPs to GTseek LLC (https://gtseek.com/) for custom locus-specific primer design.

### GT-seq test library preparation

We constructed a GT-seq test library with the previously extracted DNA samples collected in 2016 (n = 45) and 2017 (n = 59) for which otolith microchemistry analysis was previously conducted^29^. Extracted DNA was quantified with a Qubit 3.0 Fluorometer and the dsDNA High Sensitivity Assay Kit (Invitrogen). Library preparation followed the original protocol^38^, with the exception that we diluted the PCR1 product to 1:10 (10.17504/protocols.io.byvppw5n). The PCR2 product was quantified with Picogreen™ (Molecular Probes, Inc.) and each sample was normalized to a concentration of 10 ng/µL. The pooled library was purified with a MinElute PCR Purification Kit (Qiagen) and eluted into a final volume of 25µL. Test libraries were sequenced using a Mid Output Reagent Kit (300 cycles) on an Illumina MiniSeq within the Ecological and Conservation Genomics Laboratory at the University of British Columbia Okanagan.

### GT-seq genotyping and primer optimization

**Figure 2:**
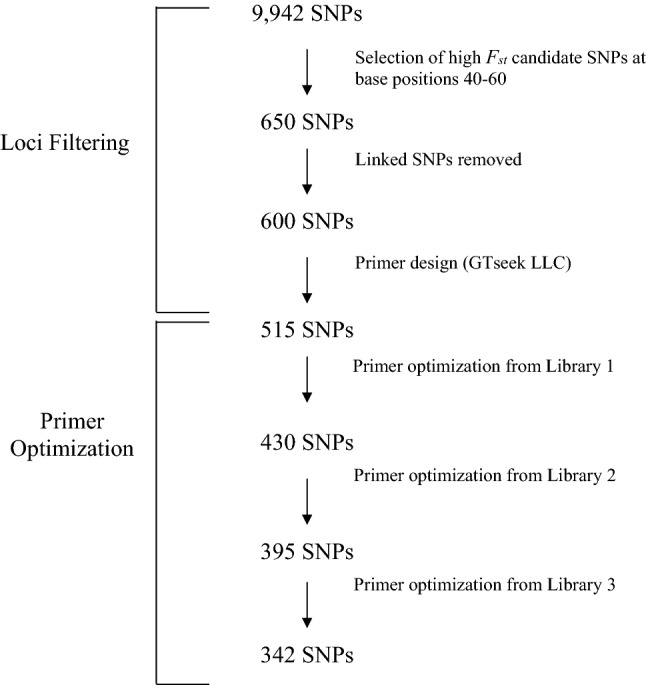
Workflow of *Oncorhynchus nerka* (sockeye salmon and kokanee) GT-seq hybridization panel design.

Demultiplexed raw sequencing files were processed with the GT-seq pipeline available on GitHub (https://github.com/GTseq/GTseq-Pipeline). We removed primers with non-specific *in silico* probes, candidates that were overrepresented (exhibiting > 2% of the raw read count), observed primer dimers, potential PCR artefacts, off-target amplification or *in silico* probe variation following previously published work^49^. A second and third test library with the same sample composition as the first test library were prepared and tested iteratively with optimized primer pools from previous libraries using the protocols detailed above for sample preparation, sequencing, and primer dropout (Fig. 2).

To compile the final dataset based on the optimized SNP panel for downstream analyses, raw sequencing files from individuals were concatenated across sequencing runs and processed with the GT-seq pipeline available on GitHub (https://github.com/GTseq/GTseq-Pipeline). We filtered out individuals with > 25% missing data using PLINK^50^. We decided on a cutoff of 25% to minimize the proportion of missing data that can negatively impact the detection of population structure, particularly with the high resolution needed to elucidate hybrid classes within a species^51^.

### Panel performance

We assessed the accuracy, efficiency and power of NEWHYBRIDS assignments with simulated individuals (n = 100) using two pools (top 300 *F*_*st*_ SNPs, optimized SNP panel) with *hybridpowercomp* as implemented in the R package *hybriddetective*^34,52^*.* Specifically, we evaluated assignment probabilities based on: (1) accuracy (correct assignments/total assignments per category); (2) efficiency (correct assignments/total number of individuals in a category); and (3) power (product of accuracy and efficiency). We also used *hybriddetective*^34,52^ to quantify error rates associated with individual assignment (top 300 *F*_*st*_ SNPs, optimized SNP panel) as follows: (1) Type I: false positive error rate (number of known pure individuals wrongly assigned to a hybrid genotype frequency class/total number of known pure individuals); and (2) Type II: false negative error rate (number of known hybrid individuals wrongly assigned to a pure genotype frequency class/total number of known hybrid individuals).

### Re-analysis of 2016–2017 samples

We genetically assigned the 2016 and 2017 sampled individuals to pure-stock or hybrid class using NEWHYBRIDS^48^, the genotypic data from the optimized SNP panel, and reference baseline genetic data from 2003 Skaha Lake kokanee and 2012 Okanagan River sockeye salmon^42^. Five genotype frequency classes (kokanee, sockeye, F_1_, F_2_, B_2_ backcross-kokanee, and B_2_ backcross-sockeye) were specified, and the analysis initiated with a burn-in period of 10,000 followed by 50,000 iterations. We applied the *s* and *z* flags to the reference sockeye salmon and kokanee populations to designate individuals of known genotype that were sampled separately from the test individuals. We then selected the maximum probability of assignment to assign individuals to the most likely class. Lastly, we compared the NEWHYBRIDS^48^ genetic assignment to pure-stock or hybrid class to the migratory history of the individuals, as previously inferred^29^ by way of otolith microchemistry analysis.

### Assignment of 2019 samples

For the new samples collected in 2019 (n = 218), DNA was extracted with a standard Chelex-based protocol in 96-well plates containing approximately 0.5 mm^2^ of tissue, 5 µl proteinase K (10 mg/ml), and 195 ul 10% Chelex solution^53^. Plates were incubated for 4 h at 55 °C to digest tissue, and then 95 °C for 15 min using an Applied Biosystems Veriti thermal cycler (Applied Biosystems, Foster City, CA, USA). Library preparation was conducted as above with the optimized SNP panel. The multiplexed pooled library was sequenced using a partial High Output Reagent Kit (300 cycles) on the Illumina MiniSeq within the Ecological and Conservation Genomics Laboratory at the University of British Columbia Okanagan. Samples with individual missing data > 25% were filtered out for downstream analyses. Individual assignment to hybrid class was conducted using NEWHYBRIDS^48^ and the same parameters as above. Fisher’s Exact Test was conducted in R to determine if proportions of hybrid class were significantly different within a sample year and between sample years.

### Morphometric analyses

One-way ANOVA and post-hoc Tukey Tests were conducted in R to determine if mean fork length for the 2019 samples was significantly different between individuals assigned to pure-stock (kokanee, sockeye salmon) and the various hybrid classes (F_1_, F_2_, B_2_ backcross-kokanee, and B_2_ backcross-sockeye).

## Results

### Initial panel ability

The simulated SNP panels provided comparable accuracy to assign individuals to hybrid class based on initial NEWHYBRIDS assignment of simulated individuals (Table 1). The top 300 *F*_*st*_ SNP panel performed best with high accuracy (> 0.98), followed by the top 600 *F*_*st*_ SNP panel (> 0.96) and the random 350 SNPs selected from the top 600 *F*_*st*_ SNPs (> 0.92).

**Table 1:** Confusion matrices of simulated individuals’ proportional assignment with NEWHYBRIDS based on data from: (A) Top 300 *F*_*st*_ SNPs; (B) Top 600 *F*_*st*_ SNPs; (C) Random 350 SNPs from the top 600 *F*_*st*_ SNPs (Random 350 *F*_*st*_); and (D) 342 final SNP panel.

A: Top 300 *F*_*st*_	Kokanee	Sockeye	F_1_	F_2_	B_2_ Kokanee	B_2_ Sockeye
Kokanee	**1.000**	0.000	0.000	0.000	0.000	0.000
Sockeye	0.000	**1.000**	0.000	0.000	0.000	0.000
F_1_	0.000	0.000	**1.000**	0.000	0.000	0.000
F_2_	0.000	0.000	0.020	**0.980**	0.000	0.000
B_2_ Kokanee	0.000	0.000	0.000	0.000	**1.000**	0.000
B_2_ Sockeye	0.000	0.000	0.000	0.000	0.000	**1.000**

B: Top 600 *F*_*st*_	Kokanee	Sockeye	F_1_	F_2_	B_2_ Kokanee	B_2_ Sockeye
Kokanee	**1.000**	0.000	0.000	0.000	0.000	0.000
Sockeye	0.000	**1.000**	0.000	0.000	0.000	0.000
F_1_	0.000	0.000	**0.960**	0.040	0.000	0.000
F_2_	0.000	0.000	0.020	**0.980**	0.000	0.000
B_2_ Kokanee	0.000	0.000	0.000	0.000	**1.000**	0.000
B_2_ Sockeye	0.000	0.000	0.000	0.000	0.000	**1.000**

C: Random 350 *F*_*st*_	Kokanee	Sockeye	F_1_	F_2_	B_2_ Kokanee	B_2_ Sockeye
Kokanee	**1.000**	0.000	0.000	0.000	0.000	0.000
Sockeye	0.000	**1.000**	0.000	0.000	0.000	0.000
F_1_	0.000	0.000	**0.970**	0.030	0.000	0.000
F_2_	0.000	0.000	0.060	**0.920**	0.020	0.000
B_2_ Kokanee	0.000	0.000	0.000	0.000	**1.000**	0.000
B_2_ Sockeye	0.000	0.000	0.000	0.010	0.000	**0.990**

D: 342 Final	Kokanee	Sockeye	F_1_	F_2_	B_2_ Kokanee	B_2_ Sockeye
Kokanee	**1.000**	0.000	0.000	0.000	0.000	0.000
Sockeye	0.000	**1.000**	0.000	0.000	0.000	0.000
F_1_	0.000	0.000	**0.950**	0.050	0.000	0.000
F_2_	0.000	0.000	0.050	**0.940**	0.000	0.010
B_2_ Kokanee	0.000	0.000	0.010	0.020	**0.970**	0.000
B_2_ Sockeye	0.000	0.000	0.000	0.020	0.000	**0.980**

Rows represent true genetic classification, with columns as assigned hybrid class. Bold values along the diagonal are correct assignment proportions. Subsets of candidate markers were tested for informativeness to assess potential primer dropout.

From the initial pool of top 600 *F*_*st*_ SNPs, primers were successfully designed for 515 SNPs after *in silico* testing. Following three rounds of multiplex amplicon sequencing and primer pool optimization, the optimized GT-seq panel consisted of 342 SNPs (Fig. 2, Supplementary Table S1). Our finalized 342 SNP panel had an accuracy of > 94% across all hybrid classes (Table 1). The lowest assignment accuracies were to F_1_ and F_2_ across all simulated panels (but all ≥ 0.92; Table 1), which is consistent with previous results^36^.

### SNP panel assignment efficacy

**Figure 3:**
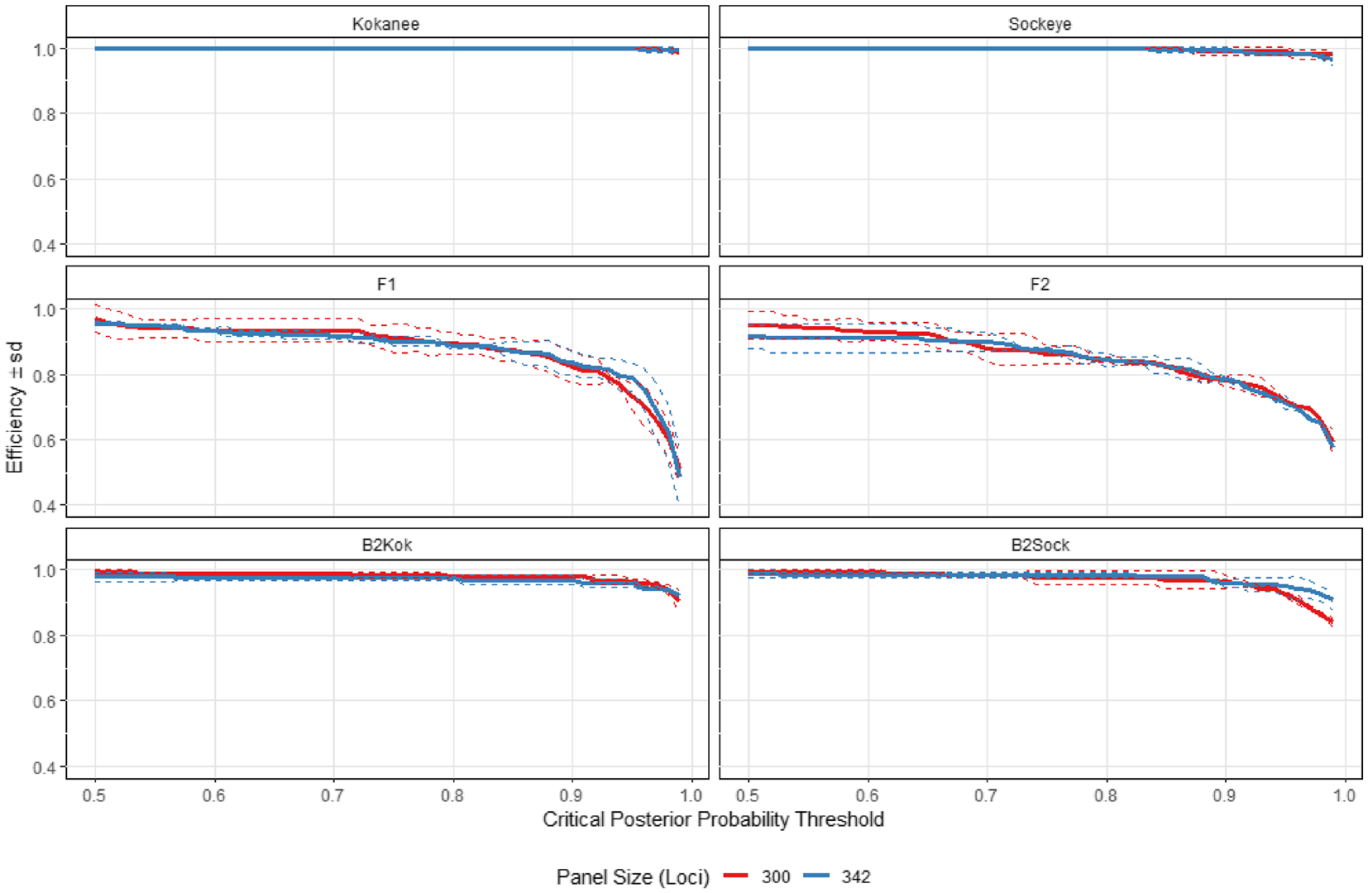
Accuracy and efficiency with simulated individuals of different hybrid classes for the top 300 *F*_*st*_ panel and the final optimized 342 SNP GT-seq panel.

The optimized 342 SNP GT-seq panel performed slightly worse than the top 300 *F*_*st*_ SNP pool, but still displayed high accuracy and efficiency in assigning simulated individuals to pure-stock and the different hybrid classes (Fig. 3). Pure-stocks and B_2_ backcrosses were detected at > 98% accuracy and > 99% efficiency at a critical posterior probability threshold of 50% (Fig. 3). F_1_ and F_2_ classes were detected at lower, but still meaningful levels, with efficiency at > 92% and accuracy at > 90% at the 50% critical probability threshold (Fig. 3). The power of assignment was comparable between the 342 SNP GT-seq panel and top 300 *F*_*st*_ SNPs where pure-stocks and B_2_ backcrosses remained stable over a wide range of probability thresholds: > 99% at a critical probability threshold of 50%, and > 98% at a threshold of 90% (Supplementary Fig. S1). However, the power of assignment for F_1_ and F_2_ classes declined in performance at higher critical posterior probability thresholds: > 90% at a critical probability threshold of 50% and > 79% at a threshold of 90% (Supplementary Fig. S1). The Type I false positive error rate and Type II false negative error rate were < 0.001% for both the 342 SNP GT-seq panel and top 300 *F*_*st*_ SNPs.

### Re-analysis of 2016–2017 Okanagan river samples

We successfully genotyped 2016 (n = 36) and 2017 (n = 55) Okanagan River spawners with the 342 SNP GT-seq panel after filtering for 25% missing data across individuals (average read depth = 220.8; genotyping rate = 90.1%). Overall, assignments to pure-stock and hybrid class were generally similar between the different marker sets, although there was a slightly higher proportion of individuals assigned to pure-stock using the 342 SNP GT-seq panel (Table 2; Supplementary Table S2). Our 342 SNP GT-seq panel further refined assignments into multiple hybrid classes for those individuals previously classified as hybrids, both in 2016 (proportion F_1_: 0.22, B_2_ backcross-kokanee: 0.03) and 2017 (proportion F_1_: 0.20, B_2_ backcross-kokanee: 0.05) (Table 2).

**Table 2:** Stock composition of spawners by sample year assessed with the TaqMan® 32 SNP assay and 342 SNP GT-seq Panel. Stock proportions indicated in parentheses.

Year	*n*	TaqMan® 32 SNP assay			342 SNP GT-seq panel					
		Kokanee	Hybrid	Sockeye	Kokanee	B_2_Kok	F_1_	F_2_	B_2_Sock	Sockeye
2016	36	16 (0.44)	12 (0.33)	8 (0.22)	16 (0.44)	1 (0.03)	8 (0.22)	0 (0.00)	0 (0.00)	11 (0.31)
2017	55	19 (0.35)	19 (0.35)	17 (0.31)	23 (0.42)	3 (0.05)	11 (0.20)	0 (0.00)	0 (0.00)	18 (0.33)
2019	202	–	–	–	115 (0.57)	24 (0.12)	16 (0.08)	4 (0.02)	19 (0.09)	24 (0.12)

A small number of assignment differences were found between the TaqMan® SNP assays^29^ and 342 SNP GT-seq panel. In 2016, four hybrids were reclassified as sockeye salmon with three instances of microchemistry conflict, where the latter results suggested that these individuals did not migrate to the ocean (Supplementary Table S2). Additionally, one sockeye salmon was genetically reclassified to F_1_ hybrid. One individual that was genetically assigned as sockeye salmon with both the 32 SNP TaqMan® assays^29^ and 342 SNP GT-seq panel had conflicting microchemistry that suggested no history of anadromy or maternal anadromy (Supplementary Table S2). Within the 2017 cohort, five individuals that were previously genetically assigned as hybrids were refined to: sockeye salmon (n = 1) and kokanee (n = 4) (Table 2; Supplementary Table S2). All microchemistry was congruent, other than one individual that was genetically assigned as sockeye salmon by both 32 SNP TaqMan® assays^29^ and the 342 SNP GT-seq panel; in this case, microchemistry suggested a resident maternal parent, but anadromous migratory history.

### Assignment of 2019 Okanagan river samples

We genotyped 2019 Okanagan River spawners (n = 202) with the 342 SNP GT-seq panel after filtering for 25% missing data across individuals (average read depth = 370.9; genotyping rate = 91.2%). The composition of the 2019 Okanagan River spawners revealed a higher proportion of kokanee (0.57; Table 2; Supplementary Table S3) and lower proportion of sockeye salmon (0.12; Table 2; Supplementary Table S3) than in 2016 and 2017 (kokanee: 0.42–0.44; sockeye salmon: 0.31–0.33; Table 2; Supplementary Table S3). In addition, the 2019 spawners contained substantially more detected backcrosses, including the first B_2_ backcross-sockeye individuals genetically identified (B_2_ backcross-kokanee: n = 24, overall proportion = 0.12; B_2_ backcross-sockeye: n = 19; overall proportion 0.09; Table 2; Supplementary Table S3). We also detected low proportions of F_2_ hybrids in 2019 (n = 4, overall proportion = 0.02) when compared to other hybrid classes.

We found significant differences in proportions among hybrid classes within a year among all sample years (2016: *P* < 0.001, χ^2^ = 45.20, df = 5; 2017: *P* < 0.001, χ^2^ = 62.68, df = 5; 2019: *P* < 0.001, χ^2^ = 292.61, df = 5). Across sample years, we found that the proportions of kokanee (*P* = 0.080, χ^2^ = 5.04, df = 2), F_2_ (*P* = 0.779, χ^2^ = 1.83, df = 2) and B_2_ backcross-kokanee (*P* = 0.140, χ^2^ = 4.35, df = 2) were not significantly different. The proportions of sockeye salmon (*P* < 0.001, χ^2^ = 16.98, df = 2), F_1_ (*P* < 0.05, χ^2^ = 10.12, df = 2), and B_2_ backcross-sockeye (*P* < 0.05, χ^2^ = 9.15, df = 2) were significantly different across sample years.

In general, sockeye salmon exhibited larger mean lengths compared with kokanee, with hybrids generally occupying an intermediate range between kokanee and sockeye salmon mean lengths (Supplementary Fig. S2; Supplementary Table S2). However, it is notable that F_2_ hybrids seem to occupy the same size distribution as B_2_ backcross-kokanee, signifying smaller body sizes with advanced hybrid classes. The mean lengths between sockeye/kokanee, F_1_/sockeye, F_2_/sockeye, B_2_ backcross-kokanee/sockeye, and B_2_ backcross-sockeye/sockeye were significantly different (Supplementary Fig. S2; Supplementary Table S2).

## Discussion

The accurate identification of advanced hybrid classes is valuable for monitoring the extent of introgression and potential fitness impacts between multiple reproductive forms of a single species or where hybridization between species is possible. SNP panels have been developed for a broad range of taxa with the ability to accurately identify hybridization between closely related species within the same genus up to the third backcross hybrid generation^54,55^. However, to our knowledge, this is the first GT-seq SNP panel with the resolution to differentiate advanced hybrid classes up to the second backcross hybrid generation across life history forms *within-species*. Our GT-seq SNP panel represents a valuable tool that may be used to examine the ongoing progression of intra-specific hybridization and potential fitness outcomes associated with an active sockeye salmon reintroduction program in the Okanagan Basin, providing information to guide on-going management strategies and offer a roadmap to other such programs for species with complex life histories.

### GT-seq panel development and evaluation

**Figure 4:**
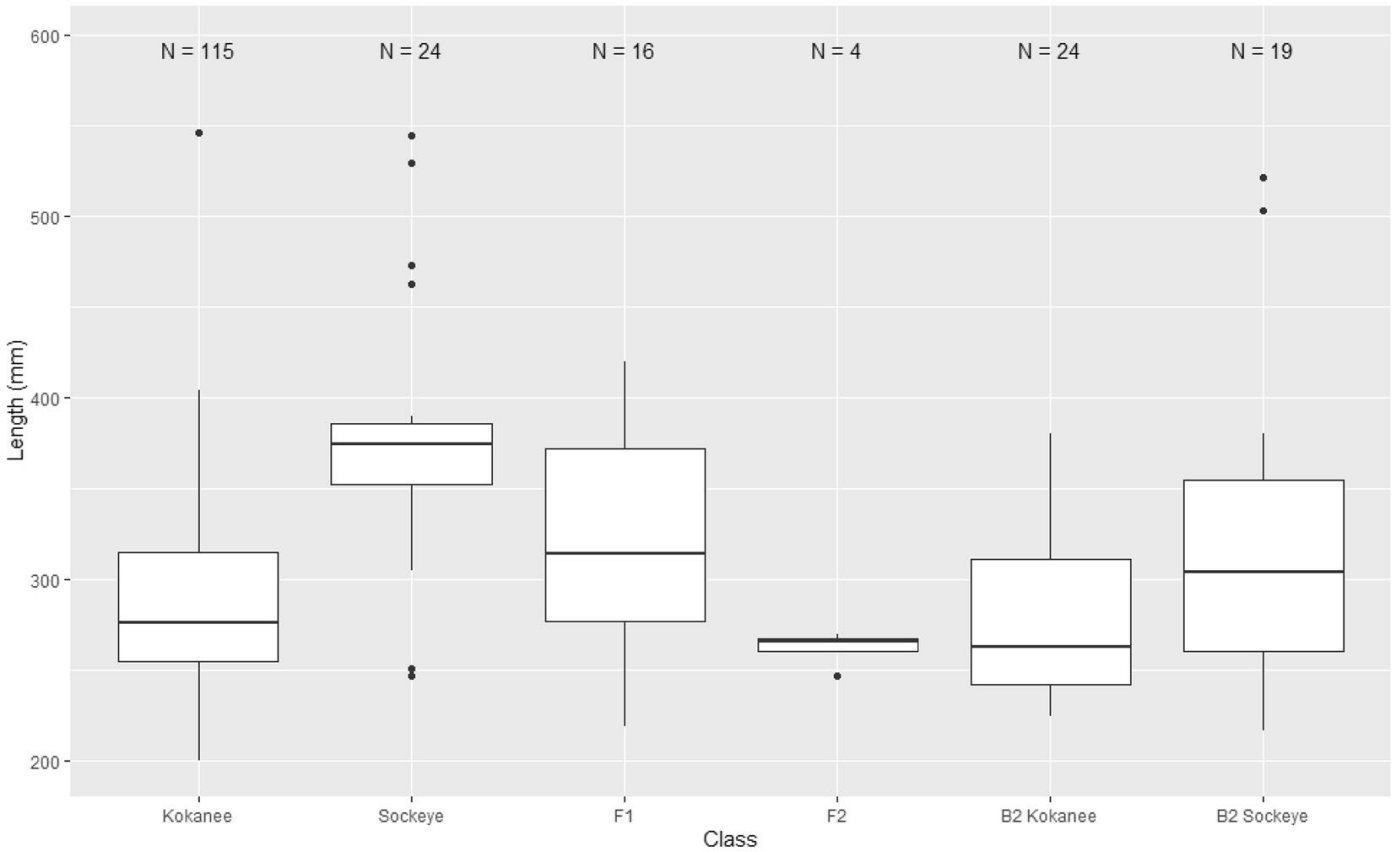
Body length of spawning *Oncorhynchus nerka* in Okanagan River Channel 2019.

Regarding initial panel development and optimization, we effectively integrated an expected level of primer drop-out within our simulation analyses to inform locus selection and provide preliminary insights on panel performance. To that end, our simulations were consistent with previous work in this system (e.g.^29,36^), and displayed high assignment accuracy across all hybrid classes (> 94%), as well as comparable panel accuracy, efficiency, and power between the top 300 *F*_*st*_ SNP pool, random 350 SNP subset of the top 600 *F*_*st*_ SNP pool, and ultimately, our optimized 342 SNP panel (Fig. 4, Supplementary Fig. S2). Subsequent error rates (< 0.001% at all levels) exhibited by the optimized 342 SNP panel reflected those of the pilot analyses, effectively streamlining the GT-seq panel preparation process and helping to ensure that panel performance met management relevant benchmarks for accuracy, efficiency, and power.

When applied to 2016 and 2017 Okanagan River spawners previously analyzed using 32 SNP TaqMan® genotyping assays and otolith microchemistry, the optimized 342 SNP panel did result in a small number of shifts in assignment of hybrids to adjacent pure-stock classes or B_2_ backcross classes. One previously classified pure-stock sockeye salmon had a refined assignment (> 99% probability) to an F_1_ hybrid. Microchemistry revealed saltwater inhabitance for the both this individual and their maternal parent; the reclassification to F_1_ hybrid suggests that that the paternal parent was likely a kokanee rather than a sockeye (Supplementary Table S2). Individuals that were refined from hybrid to pure-stock sockeye salmon (n = 2) and kokanee (n = 4) all exhibited a high probability of assignment (> 75%), consistent microchemistry, and had larger body sizes than other fish in the hybrid class (Supplementary Table S2). Reclassification of F_1_ hybrids to pure-stock sockeye salmon did not display evidence of anadromy, suggesting they may be residual sockeye salmon with anadromous parentage, but did not migrate to sea^56^. Moreover, all of these newly classified pure-stock sockeye salmon had larger body sizes than the average hybrid individual (Supplementary Table S2).

### Management implications

Sockeye salmon have been successfully re-introduced into the Okanagan Basin and comprised 41% of the *O. nerka* population by 2014^12^. The proportion of sockeye salmon in this system since that peak has significantly varied over time (12–33%), likely due to a combination of factors such as the number of spawners and fry stocked, variable ocean survival, and high water temperatures during upstream migration^57^. The re-establishment of wild spawning sockeye salmon created the possibility of hybridization with resident kokanee in later years; our results demonstrate that hybrids most recently comprised 31% of the population (2019: 8% F_1,_ 2% F_2_, 12% B_2_ backcross-kokanee, 9% B_2_ backcross-sockeye; Table 2), with advanced hybrid classes in this system present since at least 2016. Given these trends, further hybrid classes (ex: B_3_ backcross sockeye/kokanee) may develop, while the occurrence of pure-stock sockeye salmon is likely to remain due to on-going stocking programs using broodstock collected downstream from Skaha Lake.

Understanding the fitness outcomes of hybridization is important when considering the overall productivity of the system and long-term management goals. Our results and those of a previous study^29^ suggest that hybrid *O. nerka* overwhelmingly exhibit a resident life history, but can express a migratory life history with at least one F_1_ hybrid spending time in the marine environment (Supplementary Table S1). Progressive hybrid classes may also prefer a resident life history^29^ with similarities in size between F_2_ hybrids and B_2_ backcross-kokanee. However, it is still possible that backcrosses and F_2_ hybrids expressing anadromy were subject to increased mortality due to smaller size at migration or genomic incompatibilities, limiting their detection within the sampled pool of Okanagan River spawners. These trends are consistent with other salmonids, where cutthroat and steelhead trout hybrids have been found to exhibit intermediate migratory behaviors when compared to parental species that may be maladaptive to their local environment^58^. Such increased migration mortality in advanced hybrid classes expressing anadromy may signify a decrease of *O. nerka* productivity in the system. To examine the link between intermediate hybrid behavior and survival moving forward, a combination of identification and tracking methodologies may be appropriate, using passive integrated transponder (PIT) tags to mark juvenile movement, and subsequent comparison of recovered tags and genetic hybridization classes to evaluate survival and age at maturity^59^.

The population level outcomes arising from an intermediate body size in hybrids may ultimately be detrimental to the fitness of migratory hybrids compared to the larger-bodied sockeye salmon, as body size is often directly correlated with fecundity^28^. Conversely, the overall increase in body size of the resident population resulting from the presence of hybrids in the system may be advantageous for some management strategies, especially those that target increasing recreational angling quality or harvest opportunities for First Nations, as larger bodied fish generally have increased survival and are often more valued as a food source.

From a stock assessment perspective, our results emphasize that, though morphology and body size have been used historically to determine hybrid class in this system and others, advanced hybrid classes would be indistinguishable from pure-stock, with the B_2_ backcross-kokanee, F_2_ and kokanee classes having no significant mean size differences^60^. Therefore, if stock assessment programs and management goals require an estimate of stock composition, genetic tools such as GT-seq panels can be an effective enumeration method for their ability to accurately detect hybrid classes and expand sample sizes.

Here, we demonstrated the effectiveness of GT-seq for identifying advanced hybrid classes and, in this case, tracking the trajectory of sockeye salmon, kokanee, and hybrid stocks as part of a re-introduction program. Given the uncertainty in the long-term composition of the population and the range of potential outcomes and impacts on management goals, continued genetic monitoring of this system is recommended. More broadly, the GT-seq panel development workflow presented here could be applied to inform other sockeye salmon restoration initiatives in the Columbia and Fraser River systems, or in other species and systems where genetic stock identification and intra-specific hybridization are important management parameters.

## Supplementary Information

Supplementary Figure S1.

Supplementary Figure S2.

Supplementary Tables.

## Data Availability

All probe sequences and SNP genotypic data collected via GT-seq have been deposited in DRYAD (10.5061/dryad.z34tmpgg4).
