# Supplementary material for: Genotyping-in-Thousands by sequencing panel development and application for high-resolution monitoring of introgressive hybridization within sockeye salmon
Source: Sci Rep. 2022 Mar 2;12:3441. doi: 10.1038/s41598-022-07309-x (PMC8891347; doi:10.1038/s41598-022-07309-x)
Supplement: Supplementary file 1 — Supplementary Figure S1. [file 41598_2022_7309_MOESM1_ESM.docx]

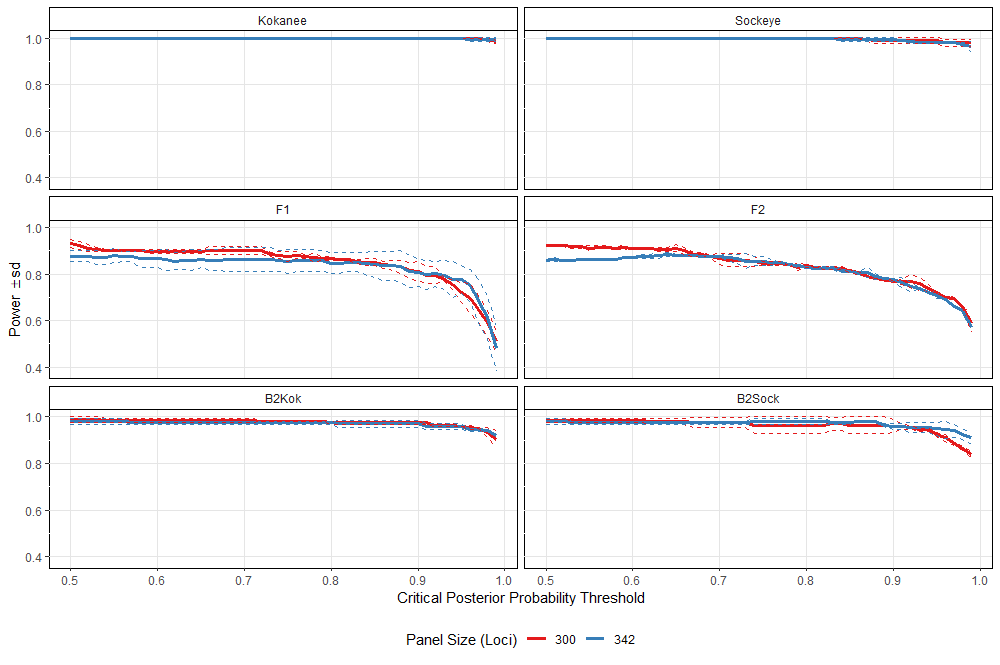


Figure S1*.* Power evaluated with simulated individuals of different hybrid classes for the top 300 *F_st_* panel and the final optimized 342 SNP GT-seq panel.
