# Supplementary material for: Genotyping-in-Thousands by sequencing panel development and application for high-resolution monitoring of introgressive hybridization within sockeye salmon
Source: Sci Rep. 2022 Mar 2;12:3441. doi: 10.1038/s41598-022-07309-x (PMC8891347; doi:10.1038/s41598-022-07309-x)
Supplement: Supplementary file 2 — Supplementary Figure S2. [file 41598_2022_7309_MOESM2_ESM.docx]

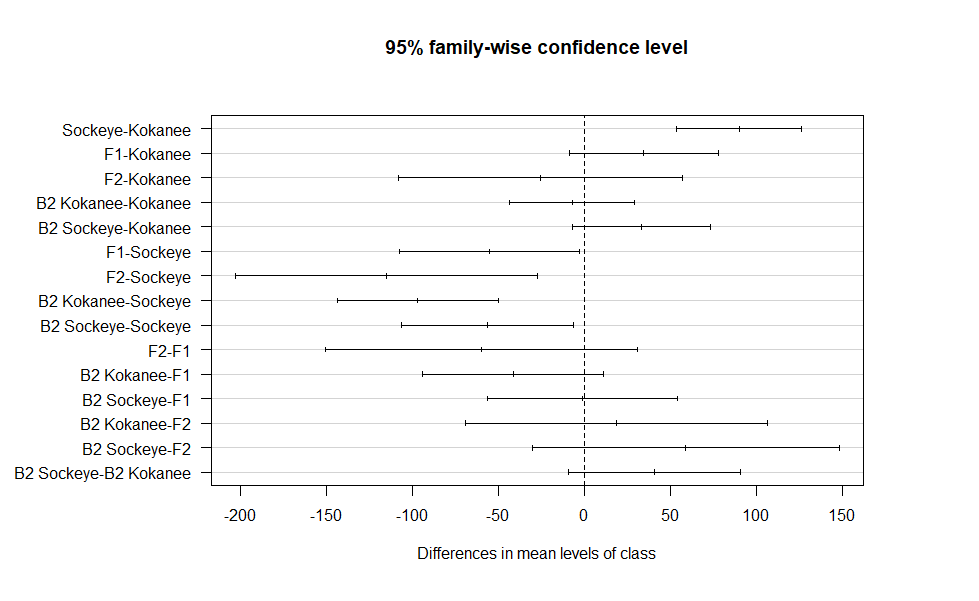
Figure S2*.* Tukey Test conducted on Okanagan River Channel 2019 for analysis of significance in body size difference between groups. Statistically significant groups did not have the 95% confidence level bar overlap with zero difference in mean levels of class.
